# Oxytocin Modulates the Cognitive Appraisal of the Own and Others Close Intimate Relationships

**DOI:** 10.3389/fnins.2019.00714

**Published:** 2019-07-16

**Authors:** Corina Aguilar-Raab, Monika Eckstein, Susanne Geracitano, Marie Prevost, Ian Gold, Markus Heinrichs, Amy Bilderbeck, Ulrike Ehlert, Beate Ditzen

**Affiliations:** ^1^Institute of Medical Psychology, Center for Psychosocial Medicine, University Hospital Heidelberg, Heidelberg, Germany; ^2^Department of Psychology, Clinical Psychology and Psychotherapy, University of Zurich, Zurich, Switzerland; ^3^Division of Social and Transcultural Psychiatry, McGill University, Montreal, QC, Canada; ^4^Department of Psychology, Laboratory for Biological and Personality Psychology, Albert-Ludwigs-University of Freiburg, Freiburg, Germany; ^5^Nuffield Department of Clinical Neurosciences, University of Oxford, Oxford, United Kingdom

**Keywords:** oxytocin, couple relationships, relationship appraisal, social cognition, repeated-measures-cross-over-design

## Abstract

Close and intimate relationships are important promoters of health. Oxytocin and its association with social cognition have been investigated in a large number of studies, especially highlighting the neuropeptide’s involvement in attachment behavior and intimate relationships. However, mixed findings on exogenous oxytocin application have led to the focus on moderators and mediators, suggesting that the effects are depended on specific factors – namely context and salience. The objective of the current study was to assess the effect of intranasal oxytocin on social appraisal of own and others’ close intimate relationship characteristics. Different characteristics of relationships, including trust or closeness, between romantic couples (unknown and own) were assessed using the Couple Appraisal Task. In a randomized controlled double-blind cross-over within subject design, *N* = 71 healthy men and women were investigated after receiving first intranasal oxytocin and 2 weeks later placebo, or vice versa. We found an oxytocin-induced increase in the positive appraisal of one’s own overall relationship characteristics but not in the evaluation of the relationship of others. The present study – one of the first of its kind administrating oxytocin in a repeated measures cross-over design – adds further evidence to the mediating role of oxytocin in social cognition, specifically with regard to romantic relationship characteristics.

## Introduction

Healthy and supportive intimate relationships promote well-being, buffer against the development of mental and physical illnesses, and improve recovery in burdened individuals (Holt-Lunstad et al., 2010; Ditzen and Heinrichs, 2014). In contrast, low-quality relationships can induce dysfunctional processes and contribute to the development of disease, if an individual is vulnerable (Fincham and Beach, 2010; Robles, 2014; Holt-Lunstad et al., 2015; Kiecolt-Glaser and Wilson, 2017).

Close positive relationships with a significant other are described by a variety of important relationship characteristics such as socialsupport (Holt-Lunstad et al., 2008), attachment and affection (Noftle and Shaver, 2006; Hadden et al., 2014), intimacy (Aron et al., 2000; Rubin and Campbell, 2012; Ferreira et al., 2014), dependency (DeHart et al., 2004; Valor-Segura et al., 2014), commitment (Stanley et al., 2010), cohesion or security (Baumeister and Leary, 1995; Olson, 2011; Kazmierczak and Blazek, 2015), as well as trust or trustworthiness (see for overall quality of relationship characteristics Schneider et al., 2011; Aguilar-Raab et al., 2015, 2018). This is also evident in theoretical models describing high functioning relationships, such as Gottman’s Sound Relationship House Theory (Gottman and Gottman, 2017) or Sternberg’s Triangular Theory of Love (Sternberg, 1986; Lemieux and Hale, 2000), the latter defining emotional, motivational, and cognitive components.

Despite the evidence of their impact on health, relatively little is known about the neurochemical mechanisms and their interconnected and complex functioning, that underpin relationship characteristics shaping social interactions with significant others.

On a neuroendocrine level, the neuropeptide and hormone oxytocin (OT) is considered to be involved in emotional and cognitive processes within intimate relationships (Hurlemann and Scheele, 2016; Feldman, 2017). On the one hand, OT is assumed to be released during partner contract (Grewen et al., 2005). On the other hand, when given exogenously, it has been shown to dampen stress-sensitive activation of the hypothalamic pituitary adrenal (HPA) axis and to enhance positive behavior during couple conflict (Ditzen et al., 2009, 2012). Furthermore, it seems to stimulate the central reward system when viewing pictures or feeling touch of the partner (Scheele et al., 2013). One functional aspect of these effects might be seen in the protection of the own established relationship via increasing the distance to potential other partners such as an attractive unacquainted person (Scheele et al., 2012). In addition, it strengthens sexual experiences with the partner (Behnia et al., 2014). Another function can be to increase the beneficial effects of partner support and touch, e.g., when experiencing acute pain (Kreuder et al., 2018). Health-promoting effects of partner contact in combination with OT are assumed to be a result of a learned association of safety experiences during the individual relationship history (Eckstein et al., 2018b).

Experimental manipulations of intranasally administered OT in association with social cognition have increased tremendously in the last two decades. These studies suggest that OT cannot be regarded as a “love-hormone” per se (Guzman et al., 2013) but instead much depends on the context (with e.g., strong social in- and out-group effects; De Dreu et al., 2010, 2011). Most importantly, the individual significance and direction of OT effects seems to depend on personal experience (Heim et al., 2008; Bertsch et al., 2013).

Intranasally administered OT has been shown to reduce the neuroendocrine response to stressful social interactions (Heinrichs et al., 2003; Ditzen et al., 2009; Zietlow et al., 2018). The administration of intranasal OT has also revealed mediation effects in the framework of social cognition and perception, such as a better ability to infer the affective state of others based on a more accurate appraisal of social cues of the eye region (Domes et al., 2007). Some findings support evidence for OT to promote qualities like generosity, cooperation, and trust (Zak et al., 2004; Kosfeld et al., 2005; Theodoridou et al., 2009). Furthermore, Fischer-Shofty et al. (2013) for example could show that OT had an overall effect on improving accurate perception of social interactions based on an Interpersonal Perception Task. On a central nervous system level, the key regions of OT effects comprise among others - the striatum, amygdala, cingulate, and insula (Winston et al., 2002; Watabe et al., 2011; Xu et al., 2012; Bethlehem et al., 2013; Scheele et al., 2013).

However, so far, findings on OT and social cognition yielded mixed and inconclusive results. For example, for effects of OT on trust, a recent review and meta-analysis summarized the available data quite critically especially in terms of variations in applied methods assessing OT, for example in peripheral bodily fluids (Nave et al., 2015): Therefore, they call for further research critically investigating the role of OT for trust-related processes, and for replicating findings and publishing controversial or null-findings.

Besides that, OT exhibits effects especially toward difficult or ambiguous items (Domes et al., 2007), and leads to higher concordant ratings for self- and other judgments (Colonnello et al., 2013). In addition, the novelty or hormonal state at prior exposure to the stimuli can play a significant role (Wallen and Rupp, 2010; Tops et al., 2013; Eckstein et al., 2018a). Shamay-Tsoory and Abu-Akel (2016) suggested that one possible mechanism to explain these findings is an influence of OT on salience and attention orientation toward social stimuli with dependence on the personal baseline characteristics such as gender, relationship status, or individual experiences. If social bonding, trust, and attachment are highly salient in individuals based on the involvement in a romantic relationship, OT can probably rather strengthen this salience of these important relationship characteristics (Scheele et al., 2012), and thereby enhance pre-existing tendencies. One underlying mechanism could be *a priori* individual differences in receptor density or sensitivity, which have been suggested in animal studies investigating local receptor distribution and sensitivity in the central nervous system (Insel and Shapiro, 1992; Walum and Young, 2018).

The potential influence of intranasally administered OT on how people perceive and evaluate important aspects of the relationship quality of others’ as well as their own close relationships, has not been fully understood so far. These processes likely form the basis of the hypothesized relevance of OT for the health-promoting effect of social relationships.

The present research aimed to investigate the influential role of OT on cognitive appraisals of romantic relationships and important relationship-defining characteristics. Using a previously published standard set of pictures and criteria (Bilderbeck et al., 2011), the question was on how OT would influence the study participants’ appraisal of their own and others’ relationships – taking into account the physical proximity (with vs. without physical contact) of the unknown couples shown on a set of photographs as potential mediator of perceived bonding (Bilderbeck et al., 2011).

To control for potential person-related factors, we applied a controlled double-blind cross-over within-subject design allowing to test order-effects (Kim et al., 2015; Eckstein et al., 2018a). Both, healthy women and men received first intranasal OT and 2 weeks later Placebo (PL), or vice versa. We hypothesized that OT – compared to PL – would lead to higher positive ratings of both other and own relationship characteristics in couples, such as intimacy or trust.

## Materials and Methods

### Participants

**FIGURE 1:**
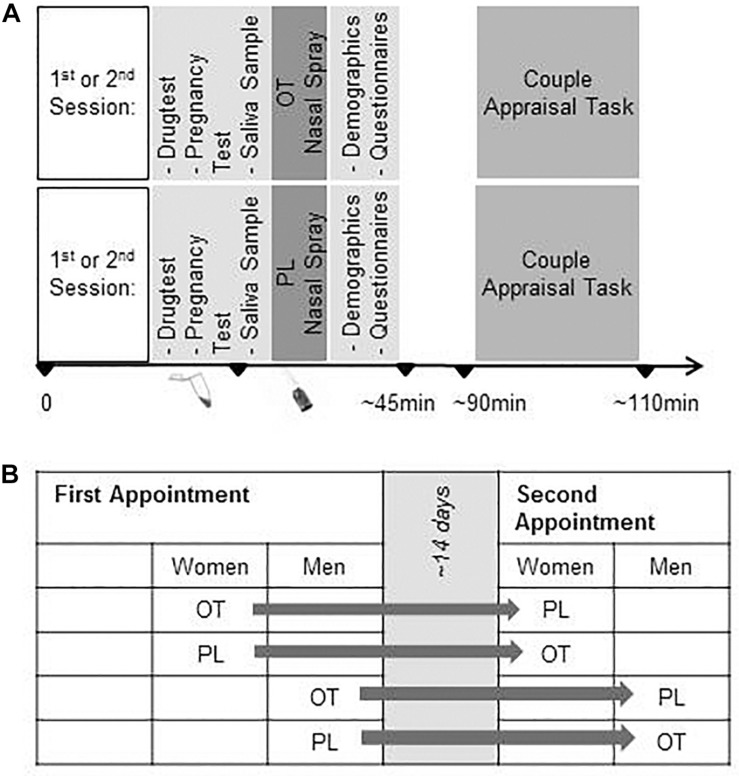
**(A)** Experimental Procedure, and **(B)** Randomized Cross-Over Design. OT, oxytocin; PL, placebo.

The study was conducted at University of Zurich, Switzerland. In a repeated-measures design, initially *N* = 84 heterosexual men and women were randomly and double blind assigned to receiving intranasal OT in the first session and PL in the second session or vice versa (see Figures 1A,B). Prior to the experimental sessions, a telephone screening was conducted to exclude participants with the following criteria: Chronic physical or mental illness, regular smoking, alcohol consumption or drug abuse, medication intake, including intake of hormonal contraceptives, and BMI above 25 or below 18. Additional exclusion criteria for women were irregular menstrual cycle (<23/>35 days), current pregnancy, and breastfeeding. All women were naturally cycling and scheduled balanced for cycle phase (50% were invited for the first session during the mid-luteal cycle phase and 50% during follicular phase according to repeated self-report and repeated monitoring).

Participants were recruited via university advertisements and public media and received either financial incentive (100 CHF) or study credits. All participants provided written informed consent before beginning with the experimental sessions. The study was approved by the local and cantonal ethics committee of the Canton of Zurich (2009/0063/5) as well as by Swissmedic, conducted in compliance with the Declaration of Helsinki and monitored from the Clinical Trials Unit (CTU) of the University of Zurich.

A total of *N* = 71 participants with *N* = 38 female and *N* = 33 male adult participants with a mean age of *M* = 26.37 (*SD* = 5.36; age range 21–45 years) were included in the final data analysis. For reasons of technical difficulties, missing data and dropout, *N* = 13 participants were previously excluded. Of the final sample, *N* = 33 (46.5%) received OT and *N* = 38 (53.5%) received PL first. *N* = 40 (56.3%) participants indicated to be single, whereas *N* = 31 (43.7%) specified to live in a romantic relationship – the latter sub-sample was used for the analysis of the Couples Appraisal Task (CAT) regarding the participants’ own relationship. This sub-sample stated to be highly satisfied with their partnership, as suggested from their scores in the Relationship Assessment Scale (seven item RAS, German version; mean score range 2.86–5.00; 4th and 7th item were recoded; higher ratings indicate higher relationship satisfaction) with *M* = 4.21 (SD = 0.11). Table 1 shows all other sample characteristics – from age for all subgroups, nationality up to annual income and others.

**TABLE 1:** Sample characteristics.

		**Both groups**			**Group 1**			**Group 2**		
		**all**	**female**	**male**	**all**	**female**	**male**	**all**	**female**	**male**
	*n*	71	38	33	33	19	14	38	19	19
Age	*M*	26.37	25.47	27.39	26.39	26.53	26.21	26.34	24.42	28.26
	*SD*	5.36	4.37	6.23	0.50	5.25	5.73	5.42	3.07	6.58
	range	21;42	21;38	21;42	21;40	21;38	21;40	21;42	21;30	21;42
Nationality	CH	78.9	71.1	87.9	78.8	78.9	78.6	78.9	63.2	94.7
	D	5.6	7.9	3.0	3.0	5.3	0	7.9	10.5	5.3
	other	15.5	21.1	9.1	18.2	15.8	21.4	13.2	26.3	0
Relationship	no	56.3	52.6	60.6	57.6	52.6	64.3	55.3	52.6	57.9
	yes	43.7	47.4	39.4	42.4	47.4	35.7	44.7	47.4	42.1
Sex. Orient.	Heterosex.	97.2	94.7	100	93.9	89.5	100	100	100	100
	Bisex.	2.8	5.3	0	6.1	10.5	0	0	0	0
Edu. Level	Primary	1.4	0	3.0	3.0	0	7.1	0	0	0
	Middle	1.4	2.6	0	3.0	5.3	0	0	0	0
	Apprenticeship	5.6	2.6	9.1	0	0	0	10.5	5.3	15.8
	Vocational	4.2	5.3	3.0	6.1	5.3	7.1	2.6	5.3	0
	Baccalaureate	49.3	50.0	48.5	48.5	42.1	57.1	50.0	57.9	42.1
	Uni. Degr.	36.6	36.8	36.4	36.4	42.1	28.6	36.8	31.6	42.1
	other	1.4	2.6	0	3.0	5.3	0	0	0	0
Job	no	40.8	34.2	48.5	45.5	36.8	57.1	36.8	31.6	42.1
	yes	59.2	65.8	51.5	54.5	63.2	42.9	63.2	68.4	57.9
Income	No income	4.2	0	9.1	0	0	0	7.9	0	15.8
	Student	63.4	65.8	60.6	72.7	68.4	78.6	55.3	63.2	47.4
	≤50.000	25.4	26.3	24.2	18.2	15.8	21.4	31.6	36.8	26.3
	≤100.000	7.0	7.9	6.1	9.1	15.8	0	5.3	0	10.5

*n, Sample size; Group 1, received OT first, PL at the second measurement point; Group 2, received PL first, OT at the second measurement point; Nationalities: CH, Switzerland, D, Germany; Sex. Orient., sexual orientation; Heterosex., heterosexual; Bisex., bisexual; Edu.Level, highest educational level; Primary, primary school; Middle, middle school; Apprenticeship, apprenticeship certificate; Vocational, vocational school-leaving certificate; Baccalaureate, baccalaureate diploma/high school graduation; Uni. Degr., university degree; Income in Swiss francs; all other characteristic values, data in percent.*

### Procedure and Tasks

The study was conducted in two identically structured experimental sessions on separate days, scheduled approx. 14 days apart (*M* = 16.47 days between assessments, SD = 10.80), between 3 – 9 pm. Prior to the investigation, participants received information about the experimental sessions along with instructions to abstain from smoking, caffeine, medication, and alcohol as well as excessive sports on the days of the investigation. At the beginning of each appointment, detailed information about the procedures and confidentiality was given and written consent was obtained. A multi drug screening (M-3-1-DT, Diagnostik Nord, Schwerin, Germany), and for women additionally, a pregnancy test was done (Evial, Inopharm, Muri near Bern, Switzerland). Participants gave a saliva sample for the assessment of gonadal hormones in order to verify the women’s menstrual cycle phase.

Subsequently, the participants self-administered either 24 IU of intranasally OT (three puffs per nostril; Syntocinon Spray, Novartis, Basel, Switzerland) or PL (containing identical ingredients except for the peptide; cantonal pharmacy of Zurich) under the supervision of the study coordinator.

The trials started 45 min after OT application and took about 20 min.

### Couple Appraisal Task

**FIGURE 2:**
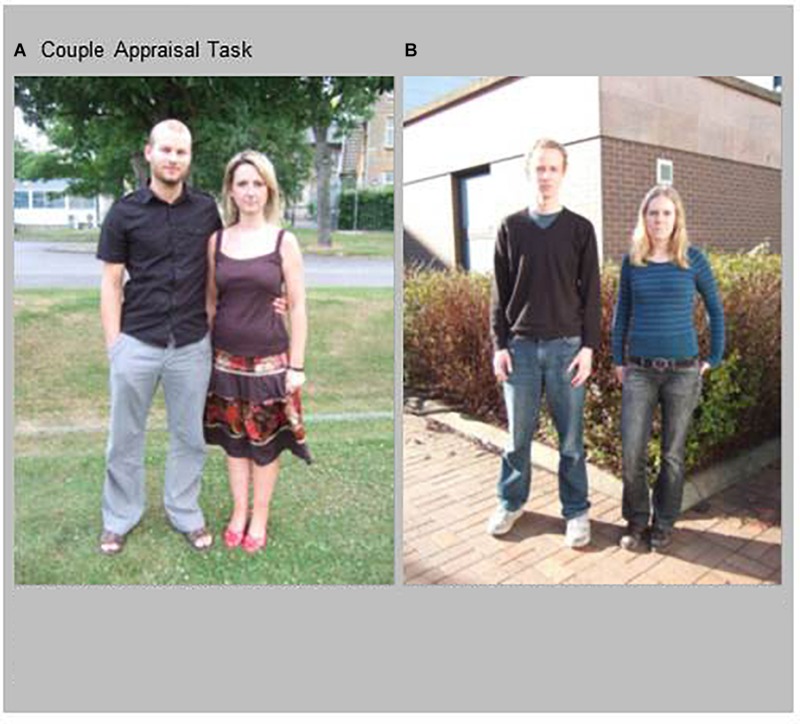
CAT Examples. Couples **(A)** with body contact, **(B)** without body contact. Written informed consent was obtained from these individuals for the publication of the two pictures.

We used the German version of the “Couple Appraisal Task,” (CAT Bilderbeck et al., 2011), to assess the evaluation of different couple specific characteristics. The CAT contains presentations of 24 pictures of heterosexual couples at different ages. Pictures were taken when couples stood outside in a neutral setting. All couples look directly into the camera and men and women show a neutral facial expression. In 12 photographs, the couples touch each other with a romantic gesture, such as holding hands or putting their arms around their shoulders (see Figure 2A), whereas in the other 12 pictures couples are standing slightly apart (see Figure 2B). The same set of pictures was used for both assessment time points.

Participants were asked to focus on each picture without time limit and to assess the following questions on a seven-point scale from 1, “not at all” to 7, “very”: (1) “How strongly do the two partners support each other?” (supportive); (2) “How strong is the intimacy between the two partners?” (intimate); (3) “How independent are the two partners from each other?” (independent); (4) “What is the commitment between the partners?” (committed); (5) “How romantic is the relationship between the two of them?” (romantic); (6) “How trustful is the relationship between the two of them?” (trusting); (7) “How certain is the relationship between the two of them?” (certainty of relationship); (8) “How well do the partners fit together?” (fitting); (9) “How well do they handle possible conflicts?” (conflict resolution); and (10) “How good is the physical relationship between the two of them?” (good physical relationship).

To investigate couple appraisal with regard to the own romantic relationship, participants, who were currently and exclusively dating, were asked to send a picture of themselves together with their partner prior to the first assessment. The same criteria as described above were required for photograph acquisition.

First the pictures of the unknown couples where presented, and if the participant was living in a romantic relationship, the picture of him-/herself together with the partner followed as one additional picture.

The ten CAT ratings of the 24 pictures of unknown couples showed good reliability (Cronbach’s Alpha = 0.96, based on *N* = 71), whereas the ten CAT ratings of the participants’ own couple pictures (based on the sub-sample of *N* = 31) yielded lower reliability (Cronbach’s Alpha = 0.76).

### Statistical Analysis

For the CAT task we calculated mean values of the ten CAT ratings of the first set of 12 pictures showing couples with body contact, and of the second set of 12 pictures showing couples without body contact. Finally we calculated mean values of all couple pictures, and for the analysis of those participants currently in a relationship (sub-group analysis) we calculated mean values of the ten CAT ratings regarding their own couple picture.

For all statistical analyses we used IBM SPSS Version 22. Repeated measures ANOVAs were used to calculate the effects of OT vs. PL (treatment factor) on the different ratings taking into account the two measurement time-points.

In line with Bilderbeck et al., 2011, we aimed to investigate differential effects of pictures with body contact and without body contact. We conducted *t*-tests or 2 × 2 repeated measures ANOVA (treatment 2 levels: OT/PL; 2 levels with pictures with body contact/without body contact). Then we exploratively ran (2 × 10) ANOVAs one for each CAT rating. These were uncorrected for multiple testing. Finally, we analyzed the subset of pictures of the own relationship. In all analyses, we added between-subject factors for sex (male vs. female) and order of assessment (OT vs. PL in first session).

## Results

The groups (receiving OT or PL first) did not differ in any of the demographic variables, which indicates successful randomization within the group assignments: gender: χ2 (1) = 0.41, *p* = 0.52; age: χ2 (20) = 16.12, *p* = 0.71; education: χ2 (6) = 7.43, *p* = 0.28; employment: χ2 (1) = 0.54, *p* = 0.46; and relationship status: χ2 (1) = 0.04, *p* = 0.85. Additionally, the groups of female and male participants did not differ with regard to age: χ2 (20) = 15.70, *p* = 0.74.

The ratings of the images of the unknown couples with and without body contact showed significant differences in a *t*-test – revealing higher ratings of the positive partnership characteristics in the couple pictures with body contact: *t*(70) = 9.12. *p* = 0.000.

CAT mean scores and standard deviations are depicted in Table 2, differentiated by OT vs. PL, by gender and by order.

**TABLE 2:** Couple Appraisal Task (CAT) Ratings^*^.

			**Unknown couples**		**Own relationship**	
category	sex	order	***n***	***M(SD)***	***n***	***M(SD)***
Oxytocin	male	Placebo → Oxytocin	19	4.60(0.49)	8	5.73(0.72)
		Oxytocin → Placebo	14	4.42(0.59)	5	6.05(0.52)
		total	33	4.52(0.53)	13	5.86(0.65)
	female	Placebo → Oxytocin	19	4.65(0.64)	9	5.90(0.79)
		Oxytocin → Placebo	19	4.65(0.59)	9	6.10(0.48)
		total	38	4.65(0.61)	18	5.94(0.64)
	total	Placebo → Oxytocin	38	4.62(0.56)	17	5.34(0.74)
		Oxytocin → Placebo	33	4.55(0.59)	14	9.09(0.48)
		total	71	4.59(0.57)	31	5.95(0.64)
Placebo	male	Placebo → Oxytocin	19	4.55(0.42)	8	5.34(0.74)
		Oxytocin → Placebo	14	4.28(0.58)	5	6.08(0.77)
		total	33	4.44(0.51)	13	5.63(0.81)
	female	Placebo → Oxytocin	19	4.68(0.63)	9	5.70(0.77)
		Oxytocin → Placebo	19	4.55(0.38)	9	5.89(0.56)
		total	38	4.62(0.52)	18	5.79(0.66)
	total	Placebo → Oxytocin	38	4.61(0.53)	17	5.53(0.76)
		Oxytocin → Placebo	33	4.44(0.49)	14	5.96(0.62)
		total	71	4.53(0.52)	31	5.72(0.72)

n, Sample size, M, Mean, SD, Standard deviation. Order: Placebo → Oxytocin, received Placebo first, Oxytocin at the second measurement point; Oxytocin → Placebo, received Oxytocin first, Placebo at the second measurement point. ^*^ overall CAT mean values, all pictures (with and without body contact).

### Appraisal of Other Couples’ Relationship Characteristics in Pictures With Versus Without Body Contact

Results of the repeated measures (2 × 2) ANOVA (treatment OT/PL, pictures with/without body contact) with the mean scores of the ten CAT ratings of the 12 couple pictures as dependent variable showed a significant main effect of body contact with higher CAT-ratings of body contact pictures: *F*(1,67) = 95.48, *p* = 0.000, η^2^ = 0.588 (this and all following η^2^ are *partial* η^2^), no interaction effect of body contact and sex *F*(1,67) = 1.37, *p* = 0.247, η^2^ = 0.020, or body contact and order *F*(1,67) = 0.06, *p* = 0.814, η^2^ = 0.001.

No significant results were found for OT treatment *F*(1,67) = 1.94, *p* = 0.169, η^2^ = 0.028, no interaction effect of treatment and sex *F*(1,67) = 0.40, *p* = 0.529, η^2^ = 0.006, and no interaction effect of treatment and order *F*(1,67) = 1.30, *p* = 0.259, η^2^ = 0.019.

### Appraisal of Other Couples’ Relationship Characteristics

Results of the repeated measures ANOVA (treatment OT/PL by time and sex) with the mean scores of the ten CAT ratings of all couple pictures (with and without body contact) as dependent variable revealed no overall main effect of OT treatment *F*(1,67) = 1.94, *p* = 0.169, η^2^ = 0.028, no interaction effect of treatment and sex *F*(1,67) = 0.40, *p* = 0.529, η^2^ = 0.006, and no interaction effect of treatment and order *F*(1,67) = 1.298, *p* = 0.259, η^2^ = 0.019.

Analog analyses of the single CAT score levels, for example for the trust ratings, showed no significant effect of OT treatment *F*(1,67) = 1.742, *p* = 0.191, η^2^ = 0.025, no interaction effect of treatment and sex *F*(1,67) = 2.546, *p* = 0.115, η^2^ = 0.037 and no interaction effect treatment and order *F*(1,67) = 0.036, *p* = 0.850, η^2^ = 0.001 (see Appendix A for the non-significant findings of all CAT scores). A significant interaction effect of treatment and order was found regarding the couple characteristic “romantic” with *F*(1,67) = 4.436, *p* = 0.039, η^2^ = 0.062. When participants first received PL, OT led to higher ratings of the couples to be more romantic.

### Appraisal of the Own Relationship’s Characteristics

**FIGURE 3:**
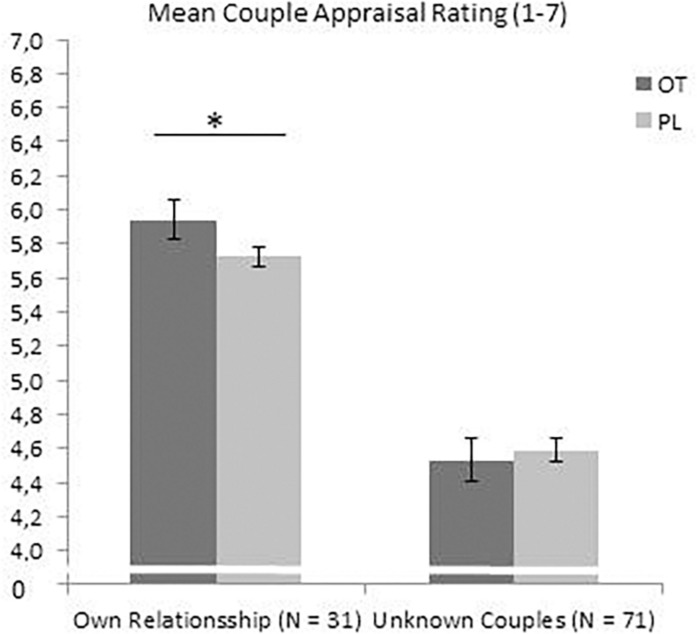
OT increased the CAT ratings toward the own relationship in the subsample of pair-bonded individuals, but not in the whole sample toward the unknown couples. ^*^p <0.05.

The analysis (repeated measures ANOVA with treatment OT/PL by time and sex) within the sub-sample of participants currently living in a romantic relationship (*N* = 31) of the mean scores of the ten CAT ratings regarding their own relationship (evaluation of their own couple picture) exhibited a main treatment effect *F*(1,27) = 4.229, *p* = 0.05, η^2^ = 0.135, suggesting higher positive couple appraisals toward the participants’ own relationship under OT, see Figure 3. No interaction effect of treatment and sex *F*(1,27) = 0.021, *p* = 0.886, η^2^ = 0.001, and no interaction effect of treatment and order *F*(1,27) = 1.14, *p* = 0.295, η^2^ = 0.041 were found. Analysis of the specific and single CAT scores showed a significant interaction effect of treatment and order on the appraisal of one’s own relationship in “conflict resolution capacities” with *F*(1,27) = 5.952, *p* = 0.02, η^2^ = 0.181. When participants received PL first, OT resulted in higher ratings of conflict resolution capacities. For all other individual CAT ratings, analysis revealed no significant results, including the trust ratings: no trust main effect of OT treatment *F*(1,27) = 0.16, *p* = 0.69, η^2^ = 0.006, no interaction effect of treatment and sex *F*(1,27) = 0.759, *p* = 0.391, η^2^ = 0.027, and no interaction effect of treatment and order *F*(1,27) = 0.028, *p* = 0.869, η^2^ = 0.001 (see Appendix A for the non-significant findings of all other CAT characteristics of one’s own relationship).

### Interaction Effects of OT/PL of Other vs. Own Relationship’s Characteristics (Based on the Sub-Sample of *n* = 31)

The repeated measures ANOVA analyses resulted in a significant within-subject effect with *F*(2,26) = 4.31, *p* = 0.024, η^2^ = 0.249, indicating higher CAT ratings under OT vs. PL for the own couple vs. the unknown couples pictures, see Figure 3.

## Discussion

The present study investigated the effects of intranasally administered OT vs. PL in a cross-over repeated measures design during a task testing for relevant and specific relationship characteristics, namely support, intimacy, independence, commitment, degree of being romantic, trust, security, fitting together, conflict resolution, and good physical relationship. Applying the Couple Appraisal Task (Bilderbeck et al., 2011) men’s and women’s evaluation of these characteristics in pictures depicting unknown couples (with and without body contact) and the participants themselves with their own partner were investigated.

While OT did not affect positive appraisals toward couple pictures unknown to the study participants – regardless of whether the couples were depicted with or without physical contact, OT significantly increased positive appraisals of these same characteristics regarding the participants’ own relationship. This effect was moderated neither by sex nor order of substance application.

These findings are in line with theoretical models and empirical data on OT’s involvement in close attachment bonds and romantic relationships, especially inducing social affiliative behavior (Taylor, 2006; Feldman, 2012, 2017). Above this, this data replicates the results from Scheele et al. (2013), where OT selectively increased positive ratings and reward-related brain activity toward the own partner vs. an unfamiliar woman. Here our data suggests that it is the overall appraisal rather than the evaluation of individual relationship characteristics, which is influenced by OT.

One underlying mechanism of these effects seems to be in the stimulation of the central nervous reward system and related dopamine activation (Walum and Young, 2018), an effect supported with data on region-specific activation in the nucleus accumbens and anterior cingulate cortex following OT administration (Scheele et al., 2013, 2015; Kreuder et al., 2017). Therefore, OT might act by increasing the rewarding aspects of the own relationship, specifically.

At the same time, the non-significant findings on the overall estimation of relationship characteristics of unknown couples shown as photographs might point to the fact that OT effects depend on individual experiences and on affiliative motivation with regard to these social stimuli. If confronted with social characteristics not linked to oneself and thereby lacking strong emotional relevance and personal involvement, OT might not necessarily influence cognition.

Above this, rather than turning social perception and interpretations toward an overall more positive view, OT was suggested to increase the stimulus materials’ salience and relevance (Shamay-Tsoory et al., 2009; Harari-Dahan and Bernstein, 2014) through influence on the amygdala and striatum, as well as the medial prefrontal cortex. In addition, it has also been suggested, that OT especially acts on salient stimuli. Our own data of another study suggests that OT increases the strong salience of negative social feedback (Eckstein et al., 2014). Another study suggested that OT increased the self-perception of positive personality traits (Cardoso et al., 2012). With regard to the present study sample, this might be of particular importance, because overall, relationship satisfaction in the current study sample was quite high (see methods section), therefore pictures of the own relationship were highly salient and very likely positively attributed. Increasing the salience of own relationship characteristics in these genuinely happy and healthy study participants might explain the specific OT effects on the participants’ own relationship characteristics. In contrast, couple appraisals regarding other – unknown – less salient couples might not be influenced by OT over this mechanism.

It might be argued, that endogenous OT mechanisms differ between singles without a partner and individuals in a couple relationship. In a relationship, there is probably more frequent endogenous stimulation of OT by social touch or sexual intimacy (de Jong et al., 2015), therefore bonded individuals may have different receptor sensitivity or density than singles, similar to what has been proposed in studies with voles (Insel and Shapiro, 1992). To date such differences cannot be tested in the living human brain.

### Limitations

The present study has some shortcomings. The CAT is an established measure (Bilderbeck et al., 2011), but the variance of perceived relationship characteristics in others vs. for the own relationship is yet to be investigated. Our data speak for a ceiling effect in favor of positive appraisals of the participant’s own positive relationship characteristics. However, the differential effect of OT on others and the own relationship characteristics might be due to the different picture samples analyzed. Using the CAT in real-time couple interaction tasks (Ditzen et al., 2007; Jarnecke et al., 2018) or study designs with ecological momentary assessments in daily life might increase the validity and generalizability of findings (c.f., Timmons et al., 2017; Doerr et al., 2018).

OT-induced increases in the CAT for the overall appraisal of one’s own relationship characteristics were not specific for gender. This missing effect might be due to the small sample size and poor statistical power. In contrast, for example, to our own previous results (Eckstein et al., 2018a), here the OT effect was dependent on order of treatment application only for the single aspect of “conflict resolution.” The missing order or carryover effect might be explained with the specific stimulus material used in this study: pictures of the own couple in comparison to pictures showing unknown couples. While in previous datasets, the stimulus material was new – and with the first presentation of these new and unacquainted stimuli there was a particularly strong OT-effect found, the own partner and photographs of the own relationship were well-known to the participants. Therefore, whether the single order-effect indicates that OT – compared to PL – had a specific impact on the evaluation of conflict resolution at the second time point, or if this is merely a spurious result due to Alpha error accumulation in multiple testing, cannot be judged with certainty and requires further exploration in the future.

Furthermore, we assessed naturally cycling women only, which does not allow for conclusions on women using hormonal contraception (Scheele et al., 2015). Gonadal hormones and opioids have been related to OT functioning (Champagne et al., 2001; Choleris et al., 2003), therefore it might be useful to systematically design future studies assessing those in order to get a full picture of the underlying complex mechanisms. Another relevant moderator, the menstrual cycle phase could not be analyzed due to small sub-group numbers and inconsistencies in self-report and endocrine markers. Still, we controlled for the cycle phase, since a recent meta-analysis shows that the endogenous oxytocin concentration in women increases or decreases depending on the respective cycle phase (Engel et al., 2019).

Moreover, it needs to be addressed that we did not measure endogenous OT levels or other neuroendocrine factors such as vasopressin, which are also important in the context of social cognition. In addition, there is the difficulty that so far hardly anything is known about daily variability of OT.

Additional factors such as genetic or epigenetic variables have also been shown to play an important role in the context of social bonds and social behavior (Jacob et al., 2007; Krueger et al., 2012; Massey et al., 2015; Feldman et al., 2016). Larger and representative biomarker studies can inform on these effects (see for example Walum et al., 2012), however, usually, these studies do not have repeated-measures behavioral data.

Moreover, due to the assumed publication bias in OT literature (Lane et al., 2016), it continues to be unknown what other tasks or effects of OT have already been tested but have not been published. Thus, the publication of null findings and unexpected results should be encouraged.

Altogether our results are in line with previous data on the modulating role of OT on couple behavior and bonding (Hurlemann and Scheele, 2016; Feldman, 2017), but also suggest that OT might not serve as a “love hormone” or rose-colored spectacles regarding romantic relationships overall. Rather, in this present sample of individuals in a genuinely happy romantic relationship, OT might have increased perceived salience and, thereby, positive appraisals of one’s own relationship. It stands to find out, whether indeed OT might serve as a possible pharmacological intervention in order to improve unhappy couple relationships. Thus, the present study adds further evidence to the mediating role of OT in social cognition and specifically estimating one’s own relationship characteristics. Future research should systematically investigate and replicate findings on neurobiological person-related factors and specific skill requirements in different tasks, relationship types and levels of relationship quality.

## Ethics Statement

All participants provided written informed consent before beginning with the experimental sessions. The study was approved by the local and cantonal ethics committee of the Canton of Zurich (2009/0063/5) as well as by the Swissmedic, conducted in compliance with the Declaration of Helsinki and monitored from the Clinical Trials Unit (CTU) of the University of Zurich.

## Author Contributions

BD, SG, MP, IG, MH, and UE designed the experiments. MP and SG conducted the experiments. AB conceptualized and tested the CAT and provided the CAT-stimuli. CA-R and ME analyzed the data. CA-R, ME, and BD wrote the manuscript.

## Conflict of Interest Statement

The authors declare that the research was conducted in the absence of any commercial or financial relationships that could be construed as a potential conflict of interest.

## References

[B1] Aguilar-Raab C. Grevenstein D. Gotthardt L. Jarczok M. N. Hunger C. Ditzen B. (2018). Changing me, changing us: relationship quality and collective efficacy as major outcomes in systemic couple therapy. *Fam. Process* 57 342–358. 10.1111/famp.12302 28657111

[B2] Aguilar-Raab C. Grevenstein D. Schweitzer J. (2015). Measuring social relationships in different social systems: the construction and validation of the evaluation of social systems (EVOS) scale. *PLoS One* 10:e0133442. 10.1371/journal.pone.0133442 26200357 PMC4511583

[B3] Aron A. Norman C. C. Aron E. N. McKenna C. Heyman R. E. (2000). Couples’ shared participation in novel and arousing activities and experienced relationship quality. *J. Pers. Soc. Psychol.* 78 273–284. 10.1037/0022-3514.78.2.273 10707334

[B4] Baumeister R. F. Leary M. R. (1995). The need to belong: desire for interpersonal attachments as a fundamental human motivation. *Psychol. Bull.* 117 497–529. 7777651

[B5] Behnia B. Heinrichs M. Bergmann W. Jung S. Germann J. Schedlowski M. (2014). Differential effects of intranasal oxytocin on sexual experiences and partner interactions in couples. *Horm. Behav.* 65 308–318. 10.1016/j.yhbeh.2014.01.009 24503174

[B6] Bertsch K. Schmidinger I. Neumann I. D. Herpertz S. C. (2013). Reduced plasma oxytocin levels in female patients with borderline personality disorder. *Horm. Behav.* 63 424–429. 10.1016/j.yhbeh.2012.11.013 23201337

[B7] Bethlehem R. A. van Honk J. Auyeung B. Baron-Cohen S. (2013). Oxytocin, brain physiology, and functional connectivity: a review of intranasal oxytocin fMRI studies. *Psychoneuroendocrinology* 38 962–974. 10.1016/j.psyneuen.2012.10.011 23159011

[B8] Bilderbeck A. C. McCabe C. Wakeley J. McGlone F. Harris T. Cowen P. J. (2011). Serotonergic activity influences the cognitive appraisal of close intimate relationships in healthy adults. *Biol. Psychiatry* 69 720–725. 10.1016/j.biopsych.2010.12.038 21396628

[B9] Cardoso C. Ellenbogen M. A. Linnen A.-M. (2012). Acute intranasal oxytocin improves positive self-perceptions of personality. *Psychopharmacology* 220 741–749. 10.1007/s00213-011-2527-6 22012170

[B10] Champagne F. Diorio J. Sharma S. Meaney M. J. (2001). Naturally occurring variations in maternal behavior in the rat are associated with differences in estrogen-inducible central oxytocin receptors. *Proc. Natl. Acad. Sci. U.S.A.* 98 12736–12741. 10.1073/pnas.221224598 11606726 PMC60123

[B11] Choleris E. Gustafsson J. -Å. Korach K. S. Muglia L. J. Pfaff D. W. Ogawa S. (2003). An estrogen-dependent four-gene micronet regulating social recognition: a study with oxytocin and estrogen receptor-α and-β knockout mice. *Proc. Natl. Acad. Sci. U.S.A.* 100 6192–6197. 10.1073/pnas.0631699100 12730370 PMC156348

[B12] Colonnello V. Chen F. S. Panksepp J. Heinrichs M. (2013). Oxytocin sharpens self-other perceptual boundary. *Psychoneuroendocrinology* 38 2996–3002. 10.1016/j.psyneuen.2013.08.010 24064220

[B13] De Dreu C. K. Greer L. L. Van Kleef G. A. Shalvi S. Handgraaf M. J. (2011). Oxytocin promotes human ethnocentrism. *Proc. Natl. Acad. Sci. U.S.A.* 108 1262–1266. 10.1073/pnas.1015316108 21220339 PMC3029708

[B14] De Dreu C. K. W. Greer L. L. Handgraaf M. J. J. Shalvi S. Van Kleef G. A. Baas M. (2010). The neuropeptide oxytocin regulates parochial altruism in intergroup conflict among humans. *Science* 328 1408–1411. 10.1126/science.1189047 20538951

[B15] de Jong T. R. Menon R. Bludau A. Grund T. Biermeier V. Klampfl S. M. (2015). Salivary oxytocin concentrations in response to running, sexual self-stimulation, breastfeeding and the TSST: the regensburg oxytocin challenge (ROC) study. *Psychoneuroendocrinology* 62 381–388. 10.1016/j.psyneuen.2015.08.027 26385109

[B16] DeHart T. Pelham B. Murray S. (2004). Implicit dependency regulation: self-esteem, relationship closeness, and implicit evaluations of close others. *Soc. Cogn.* 22 126–146. 10.1521/soco.22.1.126.30986

[B17] Ditzen B. Heinrichs M. (2014). Psychobiology of social support: the social dimension of stress buffering. *Restor Neurol. Neurosci.* 32 149–162. 10.3233/rnn-139008 23603443

[B18] Ditzen B. Nater U. M. Schaer M. La Marca R. Bodenmann G. Ehlert U. (2012). Sex-specific effects of intranasal oxytocin on autonomic nervous system and emotional responses to couple conflict. *Soc. Cogn. Affect. Neurosci.* 8 897–902. 10.1093/scan/nss083 22842905 PMC3831552

[B19] Ditzen B. Neumann I. D. Bodenmann G. von Dawans B. Turner R. A. Ehlert U. (2007). Effects of different kinds of couple interaction on cortisol and heart rate responses to stress in women. *Psychoneuroendocrinology* 32 565–574. 10.1016/j.psyneuen.2007.03.011 17499441

[B20] Ditzen B. Schaer M. Gabriel B. Bodenmann G. Ehlert U. Heinrichs M. (2009). Intranasal oxytocin increases positive communication and reduces cortisol levels during couple conflict. *Biol. Psychiatry* 65 728–731. 10.1016/j.biopsych.2008.10.011 19027101

[B21] Doerr J. M. Nater U. M. Ehlert U. Ditzen B. (2018). Co-variation of fatigue and psychobiological stress in couples’ everyday life. *Psychoneuroendocrinology* 92 135–141. 10.1016/j.psyneuen.2018.01.016 29395487

[B22] Domes G. Heinrichs M. Michel A. Berger C. Herpertz S. C. (2007). Oxytocin improves “mind-reading” in humans. *Biol. Psychiatry* 61 731–733. 10.1016/j.biopsych.2006.07.015 17137561

[B23] Eckstein M. Bamert V. Stephens S. Wallen K. Young L. Ehlert U. (2018a). w. *Soc. Neurosci.* 10.1016/S0168-1591(02)00254-X [Epub ahead of print]. 30378456 PMC6494727

[B24] Eckstein M. de Minas A. C. A. Scheele D. Kreuder A.-K. Hurlemann R. Grinevich V. (2018b). Oxytocin for learning calm and safety. *Int. J. Psychophysiol*. 136 5–14. 10.1016/j.ijpsycho.2018.06.004 29964070

[B25] Eckstein M. Scheele D. Weber K. Stoffel-Wagner B. Maier W. Hurlemann R. (2014). Oxytocin facilitates the sensation of social stress. *Hum. Brain Mapp.* 35 4741–4750. 10.1002/hbm.22508 24659430 PMC6869318

[B26] Engel S. Klusmann H. Ditzen B. Knaevelsrud C. Schumacher S. (2019). Menstrual cycle-related fluctuations in oxytocin concentrations: a systematic review and meta-analysis. *Front. Neuroendocrin.* 52 144–155. 10.1016/j.yfrne.2018.11.002 30458185

[B27] Feldman R. (2012). Oxytocin and social affiliation in humans. *Horm. Behav.* 61 380–391. 10.1016/j.yhbeh.2012.01.008 22285934

[B28] Feldman R. (2017). The neurobiology of human attachments. *Trends Cogn. Sci.* 21 80–99. 10.1016/j.tics.2016.11.007 28041836

[B29] Feldman R. Monakhov M. Pratt M. Ebstein R. P. (2016). Oxytocin pathway genes: evolutionary ancient system impacting on human affiliation, sociality, and psychopathology. *Biol. Psychiatry* 79 174–184. 10.1016/j.biopsych.2015.08.008 26392129

[B30] Ferreira L. C. Narciso I. Novo R. F. Pereira C. R. (2014). Predicting couple satisfaction: the role of differentiation of self, sexual desire and intimacy in heterosexual individuals. *Sex Relat. Ther.* 29 390–404. 10.1080/14681994.2014.957498

[B31] Fincham F. D. Beach S. R. H. (2010). Marriage in the new millennium: a decade in review. *J. Marriage Fam.* 72 630–649. 10.1111/j.1741-3737.2010.00722.x

[B32] Fischer-Shofty M. Levkovitz Y. Shamay-Tsoory S. G. (2013). Oxytocin facilitates accurate perception of competition in men and kinship in women. *Soc. Cogn. Affect. Neurosci.* 8 313–317. 10.1093/scan/nsr100 22446301 PMC3594723

[B33] Gottman J. Gottman J. (2017). The natural principles of love. *J. Fam. Theory Rev.* 9 7–26. 10.1111/jftr.12182

[B34] Grewen K. M. Girdler S. S. Amico J. Light K. C. (2005). Effects of partner support on resting oxytocin, cortisol, norepinephrine, and blood pressure before and after warm partner contact. *Psychosom. Med.* 67 531–538. 10.1097/01.psy.0000170341.88395.47 16046364

[B35] Guzman Y. F. Tronson N. C. Jovasevic V. Sato K. Guedea A. L. Mizukami H. (2013). Fear-enhancing effects of septal oxytocin receptors. *Nat. Neurosci.* 16 1185–1187. 10.1038/nn.3465 23872596 PMC3758455

[B36] Hadden B. W. Smith C. V. Webster G. D. (2014). Relationship duration moderates associations between attachment and relationship quality: meta-analytic support for the temporal adult romantic attachment model. *Pers. Soc. Psychol. Rev.* 18 42–58. 10.1177/1088868313501885 24026179

[B37] Harari-Dahan O. Bernstein A. (2014). A general approach-avoidance hypothesis of oxytocin: accounting for social and non-social effects of oxytocin. *Neurosci. Biobehav. Rev.* 47 506–519. 10.1016/j.neubiorev.2014.10.007 25454355

[B38] Heim C. Young L. J. Newport D. J. Mletzko T. Miller A. H. Nemeroff C. B. (2008). Lower CSF oxytocin concentrations in women with a history of childhood abuse. *Mol. Psychiatry* 14 954–958. 10.1038/mp.2008.112 18957940

[B39] Heinrichs M. Baumgartner T. Kirschbaum C. Ehlert U. (2003). Social support and oxytocin interact to suppress cortisol and subjective responses to psychosocial stress. *Biol. Psychiatry* 54 1389–1398. 10.1016/s0006-3223(03)00465-7 14675803

[B40] Holt-Lunstad J. Birmingham W. Jones B. Q. (2008). Is there something unique about marriage? The relative impact of marital status, relationship quality, and network social support on ambulatory blood pressure and mental health. *Ann. Behav. Med.* 35 239–244. 10.1007/s12160-008-9018-y 18347896

[B41] Holt-Lunstad J. Smith T. B. Baker M. Harris T. Stephenson D. (2015). Loneliness and social isolation as risk factors for mortality: a meta-analytic review. *Perspect. Psychol. Sci.* 10 227–237. 10.1177/1745691614568352 25910392

[B42] Holt-Lunstad J. Smith T. B. Layton J. B. (2010). Social relationships and mortality risk: a meta-analytic review. *PLoS Med.* 7:e1000316. 10.1371/journal.pmed.1000316 20668659 PMC2910600

[B43] Hurlemann R. Scheele D. (2016). Dissecting the role of oxytocin in the formation and loss of social relationships. *Biol. Psychiatry* 79 185–193. 10.1016/j.biopsych.2015.05.013 26122876

[B44] Insel T. R. Shapiro L. E. (1992). Oxytocin receptor distribution reflects social organization in monogamous and polygamous voles. *Proc. Natl. Acad. Sci. U.S.A.* 89 5981–5985. 10.1073/pnas.89.13.5981 1321430 PMC402122

[B45] Jacob S. Brune C. W. Carter C. Leventhal B. L. Lord C. Cook E. H. Jr. (2007). Association of the oxytocin receptor gene (OXTR) in caucasian children and adolescents with autism. *Neurosci. Lett.* 417 6–9. 10.1016/j.neulet.2007.02.001 17383819 PMC2705963

[B46] Jarnecke A. M. Barden E. Back S. E. Brady K. T. Flanagan J. C. (2018). Intimate partner violence moderates the association between oxytocin and reactivity to dyadic conflict among couples. *Psychiatry Res.* 270 404–411. 10.1016/j.psychres.2018.10.003 30308464 PMC6292734

[B47] Kazmierczak M. Blazek M. (2015). Attachment styles as predictors of the perception of couples’ cohesion. *Soc. Behav. Pers.* 43 1055–1056. 10.2224/sbp.2015.43.6.1055

[B48] Kiecolt-Glaser J. K. Wilson S. J. (2017). Lovesick: how couples’ relationships influence health. *Annu. Rev. Clin. Psychol.* 13 421–443. 10.1146/annurev-clinpsy-032816-045111 28301763 PMC5549103

[B49] Kim Y.-R. Eom J.-S. Yang J.-W. Kang J. Treasure J. (2015). The impact of oxytocin on food intake and emotion recognition in patients with eating disorders: a double blind single dose within-subject cross-over design. *PLoS One* 10:e0137514. 10.1371/journal.pone.0137514 26402337 PMC4581668

[B50] Kosfeld M. Heinrichs M. Zak P. J. Fischbacher U. Fehr E. (2005). Oxytocin increases trust in humans. *Nature* 435 673–676. 10.1038/nature03701 15931222

[B51] Kreuder A. K. Scheele D. Wassermann L. Wollseifer M. Stoffel-Wagner B. Lee M. R. (2017). How the brain codes intimacy: the neurobiological substrates of romantic touch. *Hum. Brain Mapp.* 38 4525–4534. 10.1002/hbm.23679 28580708 PMC6867116

[B52] Kreuder A. K. Wassermann L. Wollseifer M. Ditzen B. Eckstein M. Stoffel-Wagner B. (2018). Oxytocin enhances the pain-relieving effects of social support in romantic couples. *Hum. Brain Mapp.* 40 242–251. 10.1002/hbm.24368 30152573 PMC6865468

[B53] Krueger F. Parasuraman R. Iyengar V. Thornburg M. Weel J. Lin M. (2012). Oxytocin receptor genetic variation promotes human trust behavior. *Front. Hum. Neurosci.* 6:4. 10.3389/fnhum.2012.00004 22347177 PMC3270329

[B54] Lane A. Luminet O. Nave G. Mikolajczak M. (2016). Is there a publication bias in behavioural intranasal oxytocin research on humans? Opening the file drawer of one laboratory. *J. Neuroendocrinol.* 28. 10.1111/jne.12384 26991328

[B55] Lemieux R. Hale J. L. (2000). Intimacy, passion, and commitment among married individuals: further testing of the triangular theory of love. *Psychol. Rep.* 87 941–948. 10.2466/pr0.2000.87.3.941 11191410

[B56] Massey S. H. Estabrook R. O’Brien T. C. Pine D. S. Burns J. L. Jacob S. (2015). Preliminary evidence for the interaction of the oxytocin receptor gene (oxtr) and face processing in differentiating prenatal smoking patterns. *Neurosci. Lett.* 584 259–264. 10.1016/j.neulet.2014.10.049 25450139 PMC4267911

[B57] Nave G. Camerer C. McCullough M. (2015). Does oxytocin increase trust in humans? A critical review of research. *Perspect. Psychol. Sci.* 10 772–789. 10.1177/1745691615600138 26581735

[B58] Noftle E. E. Shaver P. R. (2006). Attachment dimensions and the big five personality traits: associations and comparative ability to predict relationship quality. *J. Res. Pers.* 40 179–208. 10.1016/j.jrp.2004.11.003

[B59] Olson D. H. (2011). FACES IV and the circumplex model: validation study. *J. Marital Fam. Ther.* 37 64–80. 10.1111/j.1752-0606.2009.00175.x 21198689

[B60] Robles T. F. (2014). Marital quality and health: implications for marriage in the 21st Century. *Curr. Dir. Psychol. Sci.* 23 427–432. 10.1177/0963721414549043 25544806 PMC4275835

[B61] Rubin H. Campbell L. (2012). Day-to-day changes in intimacy predict heightened relationship passion, sexual occurrence, and sexual satisfaction: a dyadic diary analysis. *Soc. Psychol. Personal. Sci.* 3 224–231. 10.1177/1948550611416520

[B62] Scheele D. Plota J. Stoffel-Wagner B. Maier W. Hurlemann R. (2015). Hormonal contraceptives suppress oxytocin-induced brain reward responses to the partner’s face. *Soc. Cogn. Affect. Neurosci.* 11 767–774. 10.1093/scan/nsv157 26722017 PMC4847696

[B63] Scheele D. Striepens N. Güntürkün O. Deutschländer S. Maier W. Kendrick K. M. (2012). Oxytocin modulates social distance between males and females. *J. Neurosci.* 32 16074–16079. 10.1523/JNEUROSCI.2755-12.2012 23152592 PMC6794013

[B64] Scheele D. Wille A. Kendrick K. M. Stoffel-Wagner B. Becker B. Güntürkün O. (2013). Oxytocin enhances brain reward system responses in men viewing the face of their female partner. *Proc. Natl. Acad. Sci. U.S.A.* 110 20308–20313. 10.1073/pnas.1314190110 24277856 PMC3864312

[B65] Schneider I. K. Konijn E. A. Righetti F. Rusbult C. E. (2011). A healthy dose of trust: the relationship between interpersonal trust and health. *Pers. Relatsh.* 18 668–676. 10.1111/j.1475-6811.2010.01338.x

[B66] Shamay-Tsoory S. G. Abu-Akel A. (2016). The social salience hypothesis of oxytocin. *Biol. Psychiatry* 79 194–202. 10.1016/j.biopsych.2015.07.020 26321019

[B67] Shamay-Tsoory S. G. Fischer M. Dvash J. Harari H. Perach-Bloom N. Levkovitz Y. (2009). Intranasal administration of oxytocin increases envy and schadenfreude (gloating). *Biol. Psychiatry* 66 864–870. 10.1016/j.biopsych.2009.06.009 19640508

[B68] Stanley S. M. Rhoades G. K. Whitton S. W. (2010). Commitment: functions, formation, and the securing of romantic attachment. *J. Fam. Theory Rev.* 2 243–257. 10.1111/j.1756-2589.2010.00060.x 21339829 PMC3039217

[B69] Sternberg R. J. (1986). A triangular theory of love. *Psychol. Rev.* 93 119–135. 10.1037/0033-295X.93.2.119

[B70] Taylor S. E. (2006). Tend and befriend: biobehavioral bases of ffiliation under stress. *Curr. Dir. Psychol. Sci.* 15 273–277. 10.1111/j.1467-8721.2006.00451.x

[B71] Theodoridou A. Rowe A. C. Penton-Voak I. S. Rogers P. J. (2009). Oxytocin and social perception: oxytocin increases perceived facial trustworthiness and attractiveness. *Horm. Behav.* 56 128–132. 10.1016/j.yhbeh.2009.03.019 19344725

[B72] Timmons A. C. Baucom B. R. Han S. C. Perrone L. Chaspari T. Narayanan S. S. (2017). New frontiers in ambulatory assessment: big data methods for capturing couples’ emotions, vocalizations, and physiology in daily life. *Soc. Psychol. Personal. Sci.* 8 552–563. 10.1177/1948550617709115

[B73] Tops M. Huffmeijer R. Linting M. Grewen K. Light K. Koole S. (2013). The role of oxytocin in familiarization-habituation responses to social novelty. *Front. Psychol.* 4:761. 10.3389/fpsyg.2013.00761 24151482 PMC3798760

[B74] Valor-Segura I. Expósito F. Moya M. Kluwer E. (2014). Don’t leave me: the effect of dependency and emotions in relationship conflict. *J. Appl. Soc. Psychol.* 44 579–587. 10.1111/jasp.12250

[B75] Wallen K. Rupp H. A. (2010). Women’s interest in visual sexual stimuli varies with menstrual cycle phase at first exposure and predicts later interest. *Horm. Behav.* 57 263–268. 10.1016/j.yhbeh.2009.12.005 20034495 PMC3970165

[B76] Walum H. Lichtenstein P. Neiderhiser J. M. Reiss D. Ganiban J. M. Spotts E. L. (2012). Variation in the oxytocin receptor gene is associated with pair-bonding and social behavior. *Biol. Psychiatry* 71 419–426. 10.1016/j.biopsych.2011.09.002 22015110 PMC3266986

[B77] Walum H. Young L. J. (2018). The neural mechanisms and circuitry of the pair bond. *Nat. Rev. Neurosci.* 19 643–654. 10.1038/s41583-018-0072-76 30301953 PMC6283620

[B78] Watabe M. Ban H. Yamamoto H. (2011). Judgments about others’ trustworthiness: an fMRI study. *Lett. Evol. Behav. Sci.* 2 28–32. 10.1016/j.psyneuen.2019.04.014 31015068

[B79] Winston J. S. Strange B. A. O’Doherty J. Dolan R. J. (2002). Automatic and intentional brain responses during evaluation of trustworthiness of faces. *Nat Neurosci.* 5 277–283. 10.1038/nn816 11850635

[B80] Xu X. Brown L. Aron A. Cao G. Feng T. Acevedo B. (2012). Regional brain activity during early-stage intense romantic love predicted relationship outcomes after 40 months: an fMRI assessment. *Neurosci. Lett.* 526 33–38. 10.1016/j.neulet.2012.08.004 22902992

[B81] Zak P. J. Kurzban R. Matzner W. T. (2004). The neurobiology of trust. *Ann. N. Y. Acad. Sci.* 1032 224–227. 10.1196/annals.1314.025 15677415

[B82] Zietlow A.-L. Eckstein M. Nonnenmacher N. Reck C. Schaer M. Bodenmann G. (2018). Dyadic coping and its underlying neuroendocrine mechanisms–implications for stress regulation. *Front. Psychol.* 9:2600. 10.3389/fpsyg.2018.02600 30687147 PMC6333675

