# Supplementary material for: Oxytocin Modulates the Cognitive Appraisal of the Own and Others Close Intimate Relationships
Source: Front Neurosci. 2019 Jul 16;13:714. doi: 10.3389/fnins.2019.00714 (PMC6646594; doi:10.3389/fnins.2019.00714)
Supplement: Supplementary file 1 [file Data_Sheet_1.pdf]

Appendix A

Main and interaction effects for all single CAT categories

	Unknown Couples			Own Relationship		
	F(*df*)	*p*	η^2	*F*(*df*)	*p*	η^2
CAT1						
main effect	(1,67) 2.724	.104	.039	(1,27) .325	.574	.012
treatment*sex	(1,67) .810	.371	.012	(1,27) .130	.721	.005
treatment*order	(1,67) .643	.425	.010	(1,27) .704	.409	.025
CAT2						
main effect	(1,67) 1.655	.203	.024	(1,27) .173	.681	.006
treatment*sex	(1,67) .277	.601	.004	(1,27) .432	.517	.016
treatment*order	(1,67) 1.874	.176	.027	(1,27) .719	.404	.026
CAT3						
main effect	(1,67) 2.608	.111	.037	(1,27) .406	.529	.015
treatment*sex	(1,67) 2.410	.125	.035	(1,27) 1.270	.270	.045
treatment*order	(1,67) .099	.754	.001	(1,27) 1.251	.273	.044
CAT4						
main effect	(1,67) 1.123	.293	.016	(1,27) .974	.332	.035
treatment*sex	(1,67) .006	.941	.000	(1,27) .285	.598	.010
treatment*order	(1,67) 1.569	.215	.023	(1,27) 1.885	.181	.065
CAT5						
main effect	(1,67) .179	.673	.003	(1,27) .001	.979	.000
treatment*sex	(1,67) .036	.849	.001	(1,27) 2.447	.129	.083
treatment*order	(1,67) 4.436	.039	.062	(1,27) 1.581	.219	.055
CAT6						
main effect	(1,67) 1.742	.191	.025	(1,27) .162	.690	.006
treatment*sex	(1,67) 2.546	.115	.037	(1,27) .759	.391	.027
treatment*order	(1,67) .036	.850	.001	(1,27) .028	.869	.001
CAT7						
main effect	(1,67) .327	.570	.005	(1,27) .192	.664	.007
treatment*sex	(1,67) .000	.991	.000	(1,27) .029	.866	.001
treatment*order	(1,67) .076	.784	.001	(1,27) 2.444	.130	.083
CAT8						
main effect	(1,67) .531	.469	.008	(1,27) 1.596	.217	.056
treatment*sex	(1,67) .246	.622	.004	(1,27) .015	.903	.001
treatment*order	(1,67) .464	.498	.007	(1,27) .658	.425	.024
CAT9						
main effect	(1,67) 1.250	.268	.018	(1,27) .260	.614	.010
treatment*sex	(1,67) .893	.348	.013	(1,27) 1.279	.268	.045
treatment*order	(1,67) 1.629	.206	.024	(1,27) 5.952	.022	.181
CAT10						
main effect	(1,67) 1.604	.210	.023	(1,27) 1.040	.317	.037
treatment*sex	(1,67) .025	.874	.000	(1.27) 1.442	.240	.051
treatment*order	(1,67) .313	.578	.005	(1.27) .528	.474	.019
